# Spontaneous Innovation for Future Deception in a Male Chimpanzee

**DOI:** 10.1371/journal.pone.0036782

**Published:** 2012-05-09

**Authors:** Mathias Osvath, Elin Karvonen

**Affiliations:** 1 Department of Cognitive Science, Lund University, Lund, Sweden; 2 Lund University Primate Research Station Furuvik, Furuvik, Sweden; Université Pierre et Marie Curie, France

## Abstract

**Background:**

The ability to invent means to deceive others, where the deception lies in the perceptually or contextually detached future, appears to require the coordination of sophisticated cognitive skills toward a single goal. Meanwhile innovation for a current situation has been observed in a wide range of species. Planning, on the one hand, and the social cognition required for deception on the other, have been linked to one another, both from a co-evolutionary and a neuroanatomical perspective. Innovation and deception have also been suggested to be connected in their nature of relying on novelty.

**Methodology/Principal Findings:**

We report on systematic observations suggesting innovation for future deception by a captive male chimpanzee (*Pan troglodytes*). As an extension of previously described behaviour – caching projectiles for later throwing at zoo visitors – the chimpanzee, again in advance, manufactured concealments from hay, as well as used naturally occurring concealments. All were placed near the visitors' observation area, allowing the chimpanzee to make throws before the crowd could back off. We observed what was likely the first instance of this innovation. Further observations showed that the creation of future-oriented concealments became the significantly preferred strategy. What is more, the chimpanzee appeared consistently to combine two deceptive strategies: hiding projectiles and inhibiting dominance display behaviour.

**Conclusions/Significance:**

The findings suggest that chimpanzees can represent the future behaviours of others while those others are not present, as well as take actions in the current situation towards such potential future behaviours. Importantly, the behaviour of the chimpanzee produced a future event, rather than merely prepared for an event that had been reliably re-occurring in the past. These findings might indicate that the chimpanzee recombined episodic memories in perceptual simulations.

## Introduction

We present systematic observations of a male chimpanzee who appears to have invented the use of concealments – both manufactured and naturally occurring ones – to be used for projectiles for future throwing at zoo visitors. That is, planning behaviours that produced a possibly desired outcome in the future, instead of relying on mere preparation for an upcoming situation that has been experienced before.

It has been suggested that human planning skills evolved in response to an increasingly complex social environment [1], [2]. Undoubtedly, thinking about how one's current actions will affect others' future behaviours often steers one's choices. Our long-term social predictions are arguably important in both cooperative and competitive contexts. Planning for how to deceive prey or opponents before encountering them is an effective low-cost strategy.

The ability to solve new problems or to come up with novel solutions to old problems has often been associated with innovation. Innovations for deception are prime examples of social innovations [3].

### Foresight

The theoretical roots of cognitive foresight research lie in the field of memory studies. In 1972, Tulving proposed a distinction between semantic and episodic memory [4], creating an essential framework for current animal research on foresight and memory. An easy way to distinguish them is to regard the first as *knowing*, the latter as *remembering*.

The semantic system represents general knowledge about the world. By contrast, the episodic system involves perceptual simulations from a first-person perspective. Knowing that Budapest is the capital of Hungary comes from the semantic system, but remembering the sight and smell of the fig tree in the back yard of the city's royal palace comes from the episodic system.

Tulving made a notable addition to his initial theory by making a type of consciousness – *autonoetic* (self-knowing) consciousness – a necessary correlate of the episodic system [5]. At the same time Tulving was introducing autonoetic consciousness, another hypothesis was being put forward: the episodic system provides not only memories of past events but also mental constructs of possible future ones. This hypothesis has now been confirmed in several areas, from neurocognition to child development (for review see e.g. [6], [7]). It appears as though episodic memory contributes previously experiences that are recombined into a novel construct, representing a possible future event.

**Figure 1:**
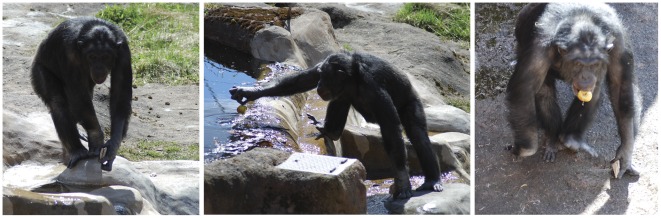
The deceptive approach. The series shows the chimpanzee when he slowly moved towards the group of visitors before releasing his projectiles. Note the two projectiles in his left hand. The picture on the left was taken 31 seconds before the throw; the central picture, where he picks up an apple from the water moat, was taken 15 seconds before; the right picture was taken 1 second before the throw. (The times are estimated from a video footage recorded at the same occasion). (Photo: Tomas Persson).

To elucidate the distinctively subjective, first-person-perspective of autonoetic consciousness, Tulving used the phrase *mental time travel*: autonoetic consciousness makes it possible to travel in time cognitively and phenomenally, to revisit or pre-visit events. Metaphorically, autonoetic consciousness provides the “inner eye” by which one “sees” past or future, perceptually simulated, events.

Animal studies face a problem: it is problematic methodologically to rely on a terminology that presupposes phenomenal consciousness. This has caused considerable quandaries over how to parsimoniously interpret the results of certain studies on planning and memory in corvines and primates [8]–[15]. Is it ever possible to know whether an animal uses an episodic system given that one has no way to probe subjective experiences? Is it therefore also valid to deny the existence of an episodic system even if behavioural and neurobiological data suggest one, just because of the lack of phenomenal insight?

It is in fact not known whether the phenomenal experience that accompanies human foresight is functional or merely an epiphenomenal byproduct of other processes. It is however roughly known which brain areas are involved in episodic operations in humans, and that those operations seem to rely partly on re-organising stored perceptual inputs (for review see e.g. [7]). In principle, those operations are empirically testable in non-humans – indeed, they have partly been studied [16]. One way to avoid arguments dependent on phenomenological access is to distinguish sensations from perceptions: sensations describe the subjective experience of events, perceptions their physical interpretation [17]. An episodic system relying on perceptual simulation does not logically entail subjective experience. However, it does presuppose (re-)organization of perceptually detached information. This is a somewhat different way to avoid the problem of subjective experience than the one taken by Clayton and colleagues [18]: instead of returning to the initial definition of episodic memories – which did not include consciousness or simulation – we propose a more neurobiologically based, but also non-phenomenal, approach, where perceptual simulations are central.

An important empirical challenge is to show whether the future-oriented behaviour in question relies on something more than mere cognitive repetition of an entire previous experience. That is, whether the animal under study can prepare for novel situations that require mentally recombining perceptual elements into new configurations, as the human episodic system allows. Such a finding for a non-human species would strongly suggest the existence of an episodic system. Many investigations and much debate have concerned the so-called Bischof-Köhler hypothesis [19]–. Suddendorf and Corballis [24] first offered the hypothesis, stating that “…animals other than humans cannot anticipate future needs or drive states and are therefore bound to a present that is defined by their current motivational state”. It does seem that an episodic system facilitates such anticipation; however, passing or failing the Bischof-Köhler “test” is not necessary, and perhaps not even sufficient, for establishing or rejecting episodic foresight in non-human animals: a certain flexibility appears just as important. (For similar ideas, see [25])

### Deception

Numerous reports of deceptive primate behaviours exist [26], [27]. Some exist for corvines as well [28]–[31]. Byrne and Whiten [32] introduced the concept of *tactical deception*, which they later elaborated on [33]. Tactical deception is a type of behavioural deception, not a morphological one as for example mimicking the colour pattern of a venomous snake. Under normal circumstances, the behaviour in question is presented “honestly”; however, in this case it is used tactically, to mislead. Consider a raven that appears to make a cache in the presence of onlookers, even though it does not empty the contents of its beak.

Of course, in many instances tactical deception can occur without the deceiver having any representation of the false knowledge states of the deceived. Such representations require that one have a so-called *Theory of Mind* [34]: an understanding of that the other's psychological state lies behind the behaviour. That skill is sometimes called *mind reading*. Theory of mind or mind reading is not required where the “reader” has associatively learned relationships of others' behavioural responses to different circumstances – or even where one can reason what one *would* have done in the situation the other is in, without assuming anything about the other's state of mind. Such exceptions to mind reading could include one's generalized experience of others' direct line of gaze, with no conceptual understanding of them as “seeing”. An example would be that when food is and has been outside the other's direct line of gaze, the other makes no attempt to take it [35]. This broader category of behaviour-predicting skills is often referred to as *behaviour reading*.

Although no single study has provided unequivocal evidence for mindreading in non-human animals, some argue that the combined weight of studies imply that at least chimpanzees and some corvines take into account the goals and perceptual perspectives of others – although maybe not their beliefs [36]. Those who reject this often argue that the studies are methodologically flawed and unable even in principle to infer mental state attribution: the results could be interpreted as reflecting no more than behaviour reading [35], [37].

### Innovation

Innovations in animals have been observed in a wide range of species [38]–[40]. Such innovation has received most attention from ecological approaches and from the perspective of its role in cultural transmission. However, it remains under-studied from a cognitive perspective, so that the underlying proximate mechanisms are neither well identified nor understood. The difficulty pinpointing the cognitive mechanisms underlying innovation is partly related to the difficulty of defining it. Innovation can be viewed either as the product (i.e., a novel behaviour pattern [39]) of or the process that results in novel behaviour [41]. Given these two perspectives, Reader and Laland [42] argue that innovations (the product) are learned behaviour patterns. It follows that innovation (the process) requires learning. This excludes from the definition mere chance behaviour or innate behavioural expressions. Reader and Laland recognize that general learning alone cannot explain innovation. They suggest a number of broad cognitive mechanisms – or behavioural processes – underlying innovation (facilitating the necessary learning): e.g., exploration, insight, creativity, and behavioural flexibility. Unfortunately, these labels are all more or less poorly understood. The cognition behind innovation remains largely uncharted.

**Figure 2:**
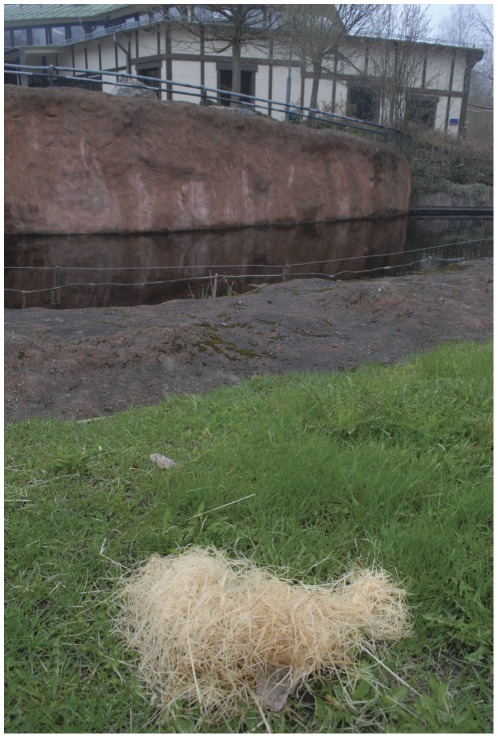
The first hay concealment made by the chimpanzee. Note the projectile in the lower part under the heap. The visible projectile above the heap was not present during the first throws. The picture was taken at the end of the day.

What is interesting given the scope of the current study is the way that innovation and deception have been linked in the context of primates' social life [3], [39]. The two skills do seem closely related: innovation can be said to occur when an existing signal or other behaviour is used in a novel way [39]; tactical deception occurs when a familiar and normally honest signal is used in a new and misleading way [33].

### Previous report on the chimpanzee of this study

In 2009 one of us (MO) reported on the projectile related behaviour of the male chimpanzee, who is also the subject in this study [23]. In 1997 the chimpanzee started to gather stones from the water moat surrounding the outside compound and storing them hours before he threw them in dominance displays at the arriving zoo visitors. The behaviour was detected after some days of unusually high number of projectiles being thrown. When cleaning the island compound, the zookeepers found five stone caches placed at the shoreline facing the visitors' area. Following days a zookeeper placed herself in a blind to observe the chimpanzee behaviour during the morning hours. He was found to retrieve stones from the moat and place them in piles. In 1998, the chimpanzee started to manufacture projectiles by breaking off loose pieces from the compound's concrete surface, and then placing them in the caches. The behaviour was observed a high number of times during the decade covered by the report. The key findings were not only that the ape prepared for future throwing when the visitors was outside his field of perception, but also that there appeared to be a dissociation between his emotional states: calm during the gathering process, agitated during the throwing sessions. These behaviours indicate foresight based on the episodic system.

Nonetheless, concerns have been raised over how the findings should be interpreted – because no detailed data is available on the chimpanzee's behaviour and circumstances at the moment when the first caches were made [43], [44]. Such information would have been valuable for the understanding of the underlying factors behind the behaviour. That said, explanations based solely on associative learning mechanisms are difficult to motivate. Even if the behaviour did start out by chance, or if initially, the chimpanzee took the stones from the water and cached them along the shore for some purpose other than throwing them later – i.e., even if he only came later to realise that they could be thrown – one still needs an explanation for the complexity of the resulting behaviour, including the time spans and the manufacturing of projectiles. One also needs to take into account the experimental results on foresighted behaviours in chimpanzees, which suggest that associative learning alone cannot explain such behaviour. It has been experimentally controlled for that chimpanzees do not merely rely on conditioning in tasks of future tool use [22], [45]. And, on the other side of the coin, it has been suggested that chimpanzees are unable to learn to bring an item intended for future exchange for food from a human, despite extensive prior reinforcement training on the item [46]. These different findings suggest that associative learning cannot *on its own* explain foresighted behaviour in chimpanzees.

To gain more detailed information we systematically studied how the projectile related behaviour starts at the beginning of a zoo visitors' season. This does not address the problem with lack of data from the behaviour's initial inception; however, it complements the earlier work and offers potential for more fine-grained insights. During the 2010 season, previously unobserved behaviours were documented, comprising both deception and innovation in relation to the chimpanzee's projectile planning activities.

## Methods

### Ethics statement

The work was carried out under the Uppsala regional ethics committee approval No C199/9. The Swedish Agricultural board (No. 31-2599/09) has approved Furuvik Zoo as a cognitive research facility on chimpanzees.

### Subject

The male chimpanzee, Santino, was born in 1978 at Munich Zoo in West Germany. At the age of five, he was transferred to Furuvik Zoo, Sweden, where he has lived ever since. Over the years, the composition of Santino's group varied, ranging between four and seven individuals of mixed sexes and ages. When Santino became the dominant male at the age of 16, there was only one other male in the group. This male died within the first year of Santino's dominance, leaving Santino as the sole male, as he has remained until the date of this study. When this study was conducted, apart from the male, the group consisted of five females, two adults, two sub-adults and one infant.

### Methodological premises

Furuvik Zoo is only open to the general public for a short season: typically June to August. The general season is in some years preceded by a shorter pre-season – usually in May – during which the only visitors are guided educational groups. This study was carried out in 2010 and the pre-season and general season followed this pattern. The division of pre- and general season governed the methods used.

Conducting a study where human bystanders are involved presents challenges: in particular, the ethics of studying a potentially dangerous behaviour. Ethically, the observer, aware of Santino's projectile-throwing behaviour, could not fail to intervene upon observing preparations for impending throws.

During the pre-season, a zoo ethologist guided the groups, and each visitor was informed about the chimpanzee's throwing behaviour. Given this, it was ethically appropriate to observe the chimpanzee's preparation of the projectiles without interference. The pre-season afforded a well-controlled setting compared to the general season, when a large number of visitors is moving around. Among other things, it was possible to make accurate observations on whether visitors were out of the chimpanzee's view. Two principal, complementary methods were used: (i) direct behavioural observations and (ii) recovery of projectiles from the compound at the end of a day. During the general season, only the latter method could be used.

**Figure 3:**
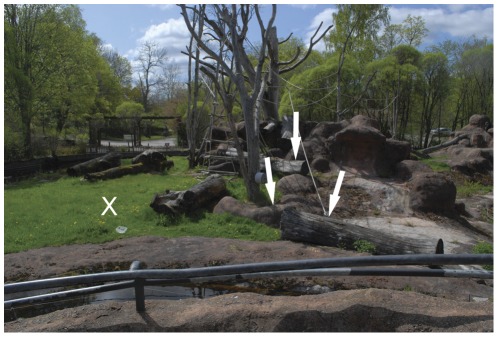
A visitor's view of the chimpanzee island. The X in the left of the picture marks the position of the first hay heap. The arrow on the left points at the protruding rock structure that was used as concealment. The other two arrows point at the two logs that also served as concealing obstacles.

### Behavioural observations

The primary goal was to address how the chimpanzee initiates his projectile-throwing behaviour at the start of the visitors' season. Therefore, behaviour sampling with continuous recording was used from the moment visitors were present during the pre-season. An observation session began the moment a visitors' group entered the vicinity of the chimpanzee compound. The session ended 30 minutes after the visitors left. Two central observational codes requires some elaboration:

*Throws* and *throw attempts* were recorded according to the position from which they were executed. It was not always possible to reliably observe the number of projectiles per throw, given the speed of the throws and the frequency with which multiple projectiles were thrown at once. Likewise it was not possible to reliably retrieve thrown projectiles, due to the dense vegetation around the compound.

A *hiding* was recorded if the observer clearly saw at least one projectile being placed behind or underneath something that would block the view. No hidings were recorded where the chimpanzee was simply active in areas that were later found to contain projectiles. This was a conservative coding, given the difficulty of seeing projectiles in the chimpanzees' closed hand. (Obviously, this code was not incorporated immediately, but only after the first observation of a hiding).

The observer needed to be out of the chimpanzee's view, during the periods when he did not have visitors. In consequence, the observer did not have an unobstructed view of the entire island: that would only have been possible with three simultaneous observers, who would have been visible to the chimpanzee. However, none of these restrictions proved problematic for recording of the essential initial behaviours.

### Recovery of projectiles

At the end of each day, remaining projectiles and concealments were documented and removed. This was the only method deployed once the general season began, and the monitoring continued for 114 days. However, Santino only engaged in projectile-related behaviour on two days of the general season.

Although none of the projectiles concealed by the hay piles originated at the place of concealment, that possibility did arise for those projectiles placed behind one of two logs, where in each case potentially loose concrete was present. The position of the projectiles might in this case then be a result of chance, rather than from intentional concealment. Therefore two types of controls were used. First, two observers independently scanned all concrete areas of the island, both visually and by probing the concrete with the side of the fist (similar to Santino's own behaviour). Second, the two observers independently examined the colour and structure of the projectiles, to judge whether they matched the pattern of the adjacent concrete.

## Results

### Initial behaviours

The primary aim of this study was to document how the projectile behaviour was initiated in a zoo season, and it turned out that the first observations yielded findings indicating intentional deception and innovation. Therefore the initial behaviours were essential and are described in detail.

The first attempt to throw projectiles in 2010 involved the first visitors of the pre-season. The attempt was preceded by typical male chimpanzee dominance display behaviour: aggressive bipedal locomotion, pilo-erection and vocalization. The projectiles were chipped off the surface layer of the concrete in the outdoor compound island immediately before they were used. The guiding zoo ethologist backed the group away before the ape could release the projectile. He consequently desisted from throwing. This pattern repeated three times in a row. When the group returned, 190 minutes later, the male made no aggressive displays. Instead he walked from the centre of the compound island toward the group, with two concrete projectiles in his hand. To the guide, his appearance did not suggest intentions of throwing. The chimpanzee even stopped and picked up an apple floating in the water from which he took a bite as he continued approaching the visitors. Just within range, he made a sudden throw at the group (see Figure 1). This behaviour fits with a category of deception referred to as *creating a neutral image*. In this case, inhibiting an aggressive intent in order to secure a close approach [3].

Following day, the chimpanzee made two further attempts, preceded by aggressive display. In both cases, the group backed away, and he desisted. When the group left, the chimpanzee were observed being active in the area of one of the logs, thereafter he brought a melon-sized heap of hay from the inside enclosure (see Figure 2). This was placed on the island, close (8 metres) to the visitors' area. Subsequently he put an unknown number of projectiles under the hay that were carried in his hand. When the group returned to the compound 60 minutes later, the chimpanzee sat beside the hay. As the group approached, without preceding display, he threw a projectile stored under the heap. Shortly after, the chimpanzee positioned himself behind the log close to another part of the visitors' area (7 metres). When the group moved into this area, he threw two stored projectiles from behind the log. No display preceded the throws. When the group left the compound again the chimpanzee was observed to cache two more projectiles under the hay pile. These were thrown, with no preceding display, 20 minutes later when the group returned to the compound. In the evening the observers recovered twelve remaining projectiles from the island, all from concrete. Out of these, seven were found in hides: one under the hay pile facing the moat, five behind the log and one under the hay outside the door to the indoor enclosure.

**Figure 4:**
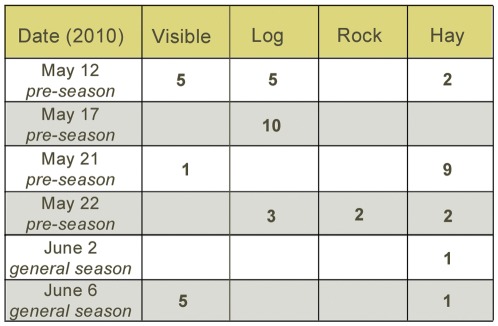
The distribution of recovered projectiles during the season. The numbers in the blocks represent the number of projectiles in each category.

A hay pile on the island, or any concealing behaviour, had not been observed previously, either by the authors of the current study or by the zookeepers. Due to the close monitoring and documentation of the chimpanzee's projectile caches since its beginning, it is close to certain that the hay hide was a first case of innovation for deception. The chimpanzee did however sometimes use hay as resting material directly outside the door to the enclosure, in a sheltered area approximately 22 meters, and out of view, from centre of the actual island. On the time of the first hay concealment the chimpanzee had taken out no such resting material, only afterwards. Although later that day this resting material also served as concealment.

### The whole zoo season

Through the course of the zoo season four hidings were directly observed as they took place (i.e. the actual projectiles were seen), always with an observer outside of the chimpanzees view. In two cases the hay was transported from the inside enclosure and placed over the projectiles, and at two occasions the projectiles were placed under the hay. In these instances the chimpanzee had first encountered a group, and cached immediately after they left. In one of these occasions he did not throw the concealed projectiles, as the group did not return. In turned out to be problematic to directly observe any unambiguous hidings behind the logs and the rock structure. Projectile oriented behaviour occurred in seven days in a period of 27 days. In all, 46 projectiles were recovered, of which 35 came from concealments. Three types of concealments were used: hay, logs (two different) and a protruding rock structure (see Figure 3 for the perspective from the visitors side on the different concealments).

Hay concealments were never placed behind the logs or the rock structure. The concealments from naturally occurring obstacles were visible to the chimpanzee but not to the visitors.

**Figure 5:**
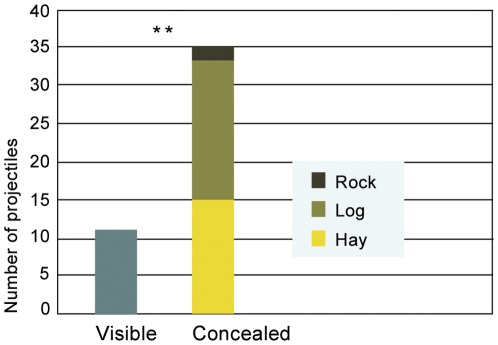
The number of visible and concealed projectiles. Throughout the season the amount of recovered concealed projectiles was significantly higher than that of the visible ones (binomial test, *P* = 0,00054).

Out of the 35 concealed recovered projectiles, 15 were placed under hay heaps (under 6 heaps; 2 “empty” heaps were also recovered), 18 were placed behind logs and 2 were placed behind a protruding rock structure (see Figure 4 for the distribution of projectiles on different dates). The non-visible projectiles were significantly more than expected by chance (binomial test, *P*<0,001) (see Figure 5). Chance level was set at 50% which is much conservative for three reasons: (1) the number of places with naturally occurring obstacles on the island is far less than 50% of the island's area; (2) the number of potential behaviours the ape can perform instead of manufacturing a hide from hay is far more than one; (3) a majority of the observed throws were made from hides, i.e. the remaining projectiles recovered from hides were fewer than the number that was actually hidden as compared to the visible caches.

The controls of the areas with loose concrete, which did not yield concealments or visible caches, revealed eight such areas in both of the independently performed controls. The distribution of the 18 projectiles behind two (the logs) out of ten possible areas with loose concrete significantly deviates from chance distribution (binomial test, *P*<0,001). The controls of colour and structure showed that at least four projectiles almost certainly did not originate from the vicinity of the concealments (only applicable for the logs; the other concealment areas did not have loose concrete). This is a highly conservative measure as the concrete is quite similar throughout the compound, and it should be understood as only a complementary control and not as the true numbers of transported projectiles.

They way the chimpanzee coupled display behaviour with throws and throwing attempts further hints at the intent with hiding the projectiles. Out of the observed throws and throwing attempts 8 were done with projectiles from hides and 8 with visible projectiles. The chimpanzee did not display a single time before throwing from a concealment, while he displayed 7 out of 8 times when the projectiles were visible; the difference between the conditions is significant (Fisher's exact test, *P* = 0,00139). The only time the chimpanzee did not display in the visible condition was in the event described in the above section. These figures should be treated with some caution, as they also include throwing *attempts* in the visible condition.

## Discussion

The likely explanation for the concealments is that they were intended for deception. Several factors converge on such an assumption: no hay concealments were ever placed behind concealing obstacles; most projectiles behind or under the concealments had been transported there; when concealed, the projectiles could be seen only from the viewpoint of the chimpanzee or not by anyone; observed throws from the concealments were never preceded by any display (suggesting the combining of two deceptive strategies); concealing behaviours were never observed when anyone was in the chimpanzee's view.

It is less clear what prompted these deceptive behaviours and the use of hay as the concealing material. One could speculate about the chimpanzee's initial throwing experience of the season, watching the people backing away. Perhaps this led him to take deceptive action, so he could release the projectiles at closer range. The first time the chimpanzee – atypically for him – was observed slowly approaching the visitors, displaying no obvious aggressive intent, before suddenly throwing projectiles at them fits well with a documented deceptive category in primates. There is no way to tell whether this was the first time he ever used this strategy. The strategy might occasionally have been used in the past. What is close to certain, however, is that there had never before been a hay concealment on the chimpanzee island, nor had projectiles ever previously been found behind naturally occurring obstacles, only as completely visible and close to the shore line.

The day the first concealments were made began as the day before, with the onlookers backing away. Those first concealments included both manufactured and naturally occurring ones. The chimpanzee was quite familiar with hay, giving him plenty of opportunities to learn its effect of blocking the view of objects; he was similarly familiar with logs. He also occasionally transported hay to a resting place just outside the door to the indoor enclosure, giving him experience of bringing hay from the inside. That said, any answer why and how he came up with the new strategy on his second day of visitors would be speculative. Interestingly, he did not start out on that second day using the deceptive strategy; his initial encounter with the visitors played out as before, and only on the second encounter did the aggression inhibition and use of concealment occur. One obvious gain from the new strategy is that the chimpanzee could use more projectiles in short succession. By combining his old strategy of gathering projectiles in advance with his new strategy of concealment and behavioural inhibition, he could extend his ability to throw stones at visitors from close range. Although, there is no way to tell whether this really was his motivation.

Both the manufacture and use of the concealments were likely premeditated. The behaviour never occurred when anyone was within the chimpanzee's view, but only after a group had been present and left: i.e., prior to their possible return. That is, it appears to have been prompted by the prior presence of visitors on those days when it occurred: the chimpanzee prepared no concealments on days when he had not previously seen visitors. This departs from the chimpanzee's previously reported behaviour, by which he typically collected projectiles in the morning before the zoo opened, on days when the zoo had visitors. That said, the earlier observations were based mainly on the general season, not on the (rare) pre-season. During the general season, visitors come every day, while during the pre-season, they arrive sporadically, several days apart (see Figure 4 for the dates of the pre-season in 2010). Taken together, the results suggest that the chimpanzee crafted a desired outcome in a perceptually detached future by acting innovatively in his current situation. Such activity *produces* a specific future event, in contrast to activity that merely prepares for a future situation as repetition of a previously experienced event. That is why the most critical finding of this study is the observation of the first instance of the concealment behaviour. This is indication of the existence of that type of perceptual simulation used by humans in certain planning tasks: a recombining of components of previously experienced events. The data further show that chimpanzees are able to plan for social situations – at least for deception – and that social planning in general is not out of reach for chimpanzees, as was suggested in a study where chimpanzees were unable to plan for future exchange with humans [46].

Do the results imply that the chimpanzee possesses a theory of mind? *Sensu stricto*, it appears as the results do not: however elaborate, the concealments could be based on the chimpanzee's understanding of line of gaze. What the behaviour does appear to show is that the chimpanzee is able to predict the behavioural responses of others not present at the time of the prediction. Mind reading is characterized as reasoning about what is not overt in behaviour: i.e., mental states. What the chimpanzee appears to be reading is likewise not overt in any behaviour (the visitors are not present). That said, the performance is possible without representing anyone else's mental states. What does seem to be a possibility is detached perceptual constructs of others' behaviours.

One means by which this might be achieved is again the episodic system, allowing the agent to simulate others in the context of a potential future situation. It has been suggested that in humans, foresight, memory, and the taking of others' viewpoints all seem to be supported by a common brain network [47]. The relevant brain structures appear to be largely shared with chimpanzees [33]. In the context of theory of mind and planning, it has been suggested that the meta-representational ability required for representing others' deviating psychological states is a prerequisite for representing *one's own* future deviating mental states and hence planning for them. The alleged lack of such an ability in non-human animals is one reason their planning is often taken to be highly restricted [1]. However, such an assumption is not necessary. When it comes to planning for your own deviating mental states it has been suggested that the perceptual construct of a potential situation plays a trick on the phylogenetically older parts of the brain: the structures governing motivation treat the construct more or less as true perceptions [48]. So, the potential future mental state, or motivation, is brought to the present and might act as a break on the motivations directed towards the current situation. When planning for potential future behaviours of others, we suggest that this could in principle also be solved by detached perceptual construct of behaviours priorly experienced under different circumstances. Then there is no need for theory-like reasoning about other's mental states, the behaviour could be “read” from the perceptual simulations (it is not necessary to represent other's mental states even for creating the constructs; a learned behavioural catalogue would suffice). What underlies the perceptual simulations of potential futures, what makes them to form, is a highly interesting question beyond the scope of speculations of this study.

The present report should be followed up by experimental investigations whether chimpanzees – and other great apes – are in general capable of planning for future deception; and whether they have the ability to form representations of future behaviours of others who are not present, given different situations. Such experiments would provide an interesting avenue for advancing the study of social cognition.

As an endnote: when observations were continued in the 2011 season, the chimpanzee did not cache or throw a single projectile. He had suffered a hip injury at the beginning of the season and was both generally slowed down and reluctant to leave his indoor enclosure. By the middle of the season, at which point he had healed, he showed no inclination to throw stones. This is consistent with the pattern in the present and previous study, in which his projectile-related behaviour was found to stop sometime before the middle of the season.

## References

[1] Suddendorf T, Corballis MC (2007). The evolution of foresight: What is mental time travel and is it unique to humans?. Behav Brain Sci.

[2] Barrett L, Henzi P, Dunbar R (2003). Primate cognition: from ‘what now?’ to ‘what if?’.. Trends Cogn Sci.

[3] Byrne RW, Reader SM, Laland KN (2003). Novelty in deceit.. Animal Innovation.

[4] Tulving E (1972). Episodic and semantic memory.. Tulving E, Donaldson W (eds) Organization of memory.

[5] Tulving E (1985). Memory and consciousness.. Canadian Psych.

[6] Suddendorf T (2010). Episodic memory verus episodic foresight: similarities and differences.. Cogn Sci.

[7] Szpunar KK (2010). Episodic future thought: An emerging concept.. Perspect Psychol Sci.

[8] Clayton NS, Bussey TJ, Emery NJ, Dickinson A (2003). Prometheus to Proust: the case for behavioural criteria for ‘mental time travel’.. Trends Cogn Sci.

[9] Suddendorf T, Busby J (2003). Like it or not? The mental time travel debate: reply to Clayton et al.. Trends Cogn Sci.

[10] Suddendorf T, Corballis MC (2008). New evidence for animal foresight?. Anim Behav.

[11] Clayton NS, Correia SPC, Raby CR, Alexis DM, Emery NJ, Dickinson A (2008). Response to Suddendorf & Corballis (2008): in defence of animal foresight.. Anim Behav.

[12] Suddendorf T, Corballis MC, Collier-Baker E (2009). How great is great ape foresight?. Anim Cogn.

[13] Osvath M (2010). Great ape foresight is looking great. Anim.. Cogn.

[14] Osvath M, Raby CR, Clayton NS (2010). What should be compared in comparative mental time travel?. Trends Cogn Sci.

[15] Roberts WA, Feeney MC (2010). Temporal sequencing is essential to future planning: response to Osvath, Raby and Clayton.. Trends Cogn Sci.

[16] Rilling JK, Barks SK, Parr LA, Preuss TM, Faber TL (2007). A comparison of resting-state brain activity in humans and chimpanzees.. Proc Natl Acad Sci USA.

[17] Humphrey N (1992). A history of the mind..

[18] Clayton NS, Bussey TJ, Dickinson A (2003). Can animals recall the past and plan for the future?. Nat Rev Neurosci.

[19] Naqshbandi M, Roberts WA (2006). Anticipation of future events in squirrel monkeys (Saimiri sciureus) and rats (Rattus norvegicus): tests of the Bischof-Kohler hypothesis.. J Comp Psychol.

[20] Raby CR, Alexis DM, Clayton NS (2007). Planning for the future by western scrub-jays.. Nature.

[21] Correia SPC, Dickinson A, Clayton NS (2007). Western scrub-jays anticipate future needs independently of their current motivational state.. Curr Biol.

[22] Osvath M, Osvath H (2008). Chimpanzee (*Pan troglodytes*) and orangutan (*Pongo abelii*) forethought: self-control and pre-experience in the face of future tool use.. Anim Cogn.

[23] Osvath M (2009). Spontaneous planning for future stone throwing by a male chimpanzee.. Curr Biol.

[24] Suddendorf T, Corballis MC (1997). Mental time travel and the evolution of the human mind.. Genet Soc Gen Psychol Monogr.

[25] Raby CR, Clayton NS (2009). Prospective cognition in animals.. Behav Proc.

[26] Byrne RW, Whiten A (1990). Tactical deception in primates: the 1990 database.. Primate Rep.

[27] de Waal FBM (1992). Intentional deception in primates.. Evol Anthropol.

[28] Bugnyar T, Kotrschal K (2002). Observational learning and the raiding of food caches in ravens, *Covus Corax*: is it ‘tactical’ deception?. Anim Behav.

[29] Bugnyar T, Kotrschal K (2004). Leading a conspecific away from food in ravens (*Corvus corax*)?. Anim Cogn.

[30] Bugnyar T, Heinrich B (2006). Pilfering ravens, *Corvus corax*, adjust their behaviour to social context and identity of competitors.. Anim Cogn.

[31] Dally JM, Emery NJ, Clayton NS (2005). Cache protection strategies by western scrub-jays, *Aphelocoma californica*: implications for social cognition.. Anim Behav.

[32] Byrne RW, Whiten A (1985). Tactical deception of familiar individuals in baboons (*Papio ursinus*).. Anim Behav.

[33] Whiten A, Byrne RW (1988). Tactical deception in primates.. Behav Brain Sci.

[34] Premack D, Woodruff G (1978). Does the chimpanzee have a theory of mind?. Behav Brain Sci.

[35] Lurz RW (2011). Mindreading animals..

[36] Call J, Tomasello M (2008). Does the chimpanzee have a theory of mind? 30 years later.. Trends Cogn Sci.

[37] Penn DC, Povinelli DJ (2007). On the lack of evidence that non-human animals possess anything remotely resembling a “theory of mind”.. Phil Trans R Soc B.

[38] Kawai M (1965). Newly-aquired pre-cultural behavior of the natural troop of Japanese monkeys on Koshima Islet.. Primates.

[39] Kummer H, Goodall J (1985). Conditions of innovative behaviour in primates.. Phil Trans R Soc B.

[40] Lefebvre L, Whittle P, Lascaris E, Finkelstein A (1997). Feeding innovations and forebrain size in birds.. Anim Behav.

[41] Lee P (1991). Adaptations to environmental change: An evolutionary perspective.. Box HO.

[42] Reader SM, Laland KN (2003). Animal innovation: an introduction.. Reader SM, Laland KN.

[43] Shettleworth SJ (2010). Clever animals and killjoy explanations in comparative psychology.. Trends Cog Sci.

[44] Suddendorf T, Corballis MC (2010). Behavioural evidence for mental time travel in nonhuman animals.. Behav Brain Res.

[45] Mulcahy NJ, Call J (2006). Apes save tools for future use.. Science.

[46] Dufour V, Sterck EHM (2008). Chimpanzees fail to plan in an exchange task but succeed in a tool-using procedure.. Behav Proc.

[47] Buckner RL, Carroll DC (2007). Self-projection and the brain.. Trends Cogn Sci.

[48] Boyer P (2008). Evolutionary economics of mental time travel?. Trends Cog Sci.

